# Cardiovascular magnetic resonance imaging for the assessment of cardiac inflammation and injury following prolonged exercise

**DOI:** 10.1186/1532-429X-11-S1-O75

**Published:** 2009-01-28

**Authors:** Rory O' Hanlon, Gregory P Whyte, Gillan Smith, Francisco Alpendurada, Joyce Wong, Matthew Wilsonl, David Oxborough, Richard Godfrey, Keith George, Annette Dahl, David Gaze, Dudley J Pennell, Sanjay K Prasad

**Affiliations:** 1 Royal Brompton Hospital, London, UK; 2 Institute for Sport and Exercise Science, Liverpool, UK; 3 University of Wolverhampton, Wolverhampton, UK; 4 University of Leeds, Leeds, UK; 5 Brunel University, London, UK; 6 Liverpool John Moores University, Liverpool, UK; 7 St George's Hospital, London, UK

**Keywords:** Cardiovascular Magnetic Resonance, Delayed Enhancement, Myocardial Oedema, Acute Myocardial Inflammation, Stir Imaging

## Introduction

Acute bouts of ultra-endurance exercise may be deleterious for cardiac structure and function as demonstrated by a reduction in diastolic and systolic function concomitant, and elevations in humoral markers of cardiac myocyte damage above acute myocardial infarction cut-off levels – most notably reflected by an elevation in troponin levels. A possible mechanism of biomarker release may be secondary to acute myocardial inflammation. CMR is established as the imaging modality of choice to visualize myocardial inflammation and fibrosis using STIR (short tau inversion recovery) imaging and early/delayed enhancement imaging following intravenous contrast administration. The relationship between ultraendurance exercise, biomarker elevation and CMR imaging for inflammation and fibrosis has not been studied before.

## Aims

We proposed that acute ultra endurance exercise in moderately trained athletes leading to elevation of cardiac troponin is associated with CMR detectable myocardial inflammation

## Methods

We performed CMR in 18 male athletes of moderate to high fitness levels 24 hrs pre and 6 hours post a marathon run. Each scan was performed by the same operator on a 1.5 T Siemens Avanto scanner using a 4 channel body array coil. Myocardial structure and function was assessed using breathhold SSFP cine imaging in long and short axis views. The presence of myocardial inflammation and oedema was assessed using STIR imaging and a 3–4 minute spin echo sequence immediately post 0.1 mmol intravenous gadolinium-DTPA (Magnavist, Schering, Germany) given using an automated injector, to assess relative gadolinium enhancement. Inversion recovery segmented-FLASH imaging with TI adjusted to null normal myocardium to highlight regions of fibrosis was used to image delayed enhancement. Each subject had bloods drawn for TnI, NT-proBNP, and CRP at baseline, immediately post and 6 hours after the run. Each CMR scan was analyzed by a blinded observer for wall thickness per segment, volumes and function, myocardial oedema using customized software (CMRTools, London, UK). Delayed enhancement images were analysed using Medis software (MASS Medis, Leiden) using a full width half maximum technique.

## Results

All 18 subjects completed the study protocol and none complained of any cardiovascular symptoms. Biventricular volumes, stroke volume, ejection fraction, and mass were unchanged pre and 6 hrs post marathon. The majority of subjects were found to have a rise in BTproBNP levels immediately and 6 hours after the race as well as elevations in TnI above the level of cut off for myocardial infarction (P = 0.001). There were no focal regions of visual signal increase on the STIR images in any of the 18 subjects. Global myocardial oedema was predefined using a cut off ratio of 1.9 comparing SI of myocardium to skeletal muscle on STIR imaging, and a relative 45% increase in the SI myocardium/skeletal muscle immediately post intravenous gadolinium on relative gadolinium enhancement imaging (rGE). No subject reached these cut off values. None had any visual myocardial fibrosis on late enhancement imaging or using automated software (MASS, Medis, Leiden).

## Conclusion

Serum markers of myocardial cell damage post ultra endurance exercise are not associated with CMR detectable levels of myocardial oedema, inflammation or scarring. These findings suggest lower degrees of myocardial damage than that normally sustained in patients with acute myocardial infarction or myocarditis, inspite of similar levels of troponin elevation.

